# The Dangers of Acetaminophen for Neurodevelopment Outweigh Scant Evidence for Long-Term Benefits

**DOI:** 10.3390/children11010044

**Published:** 2023-12-29

**Authors:** William Parker, Lauren G. Anderson, John P. Jones, Rachel Anderson, Lauren Williamson, Dillan Bono-Lunn, Zacharoula Konsoula

**Affiliations:** 1 Department of Psychology and Neuroscience, University of North Carolina, Chapel Hill, NC 27599, USA; 2 WPLab, Inc., Durham, NC 27707, USA; 3 Department of Surgery, Duke University Medical Center, Durham, NC 27710, USA; 4 Department of Biological Sciences, Northern Kentucky University, Highland Heights, KY 41099, USA; williamsol6@nku.edu; 5 Department of Public Policy, University of North Carolina, Chapel Hill, NC 27599, USA; dbonolunn@elon.edu

**Keywords:** acetaminophen, autism, fever, pain, paracetamol, postnatal, prenatal

## Abstract

Based on available data that include approximately 20 lines of evidence from studies in laboratory animal models, observations in humans, correlations in time, and pharmacological/toxicological considerations, it has been concluded without reasonable doubt and with no evidence to the contrary that exposure of susceptible babies and children to acetaminophen (paracetamol) induces many, if not most, cases of autism spectrum disorder (ASD). However, the relative number of cases of ASD that might be induced by acetaminophen has not yet been estimated. Here, we examine a variety of evidence, including the acetaminophen-induced reduction of social awareness in adults, the prevalence of ASD through time, and crude estimates of the relative number of ASD cases induced by acetaminophen during various periods of neurodevelopment. We conclude that the very early postpartum period poses the greatest risk for acetaminophen-induced ASD, and that nearly ubiquitous use of acetaminophen during early development could conceivably be responsible for the induction in the vast majority, perhaps 90% or more, of all cases of ASD. Despite over a decade of accumulating evidence that acetaminophen is harmful for neurodevelopment, numerous studies demonstrate that acetaminophen is frequently administered to children in excess of currently approved amounts and under conditions in which it provides no benefit. Further, studies have failed to demonstrate long-term benefits of acetaminophen for the pediatric population, leaving no valid rationale for continued use of the drug in that population given its risks to neurodevelopment.

## 1. Introduction

We have recently reviewed mounting evidence that acetaminophen (paracetamol) use in susceptible babies and children is associated with the development of autism spectrum disorder (ASD) and other neurodevelopmental disorders [1,2]. Based on approximately 20 lines of evidence, we previously concluded without reasonable doubt and with no evidence to the contrary that acetaminophen administration in susceptible babies and children is a causative agent for the induction of many, if not most, cases of ASD [1,2]. The conclusion is based on (a) studies in laboratory animal models [3,4,5,6,7,8], (b) understanding of the pharmacological mechanisms associated with acetaminophen toxicity [9], (c) connections between ASD, acetaminophen exposure, and human activities such as vaccination [10] and circumcision [11], (d) associations between acetaminophen administration and ASD during the later stages of pregnancy [12] and in early childhood [10,13], and (e) associations between acetaminophen use and ASD through time [9,14].

**Figure 1:**
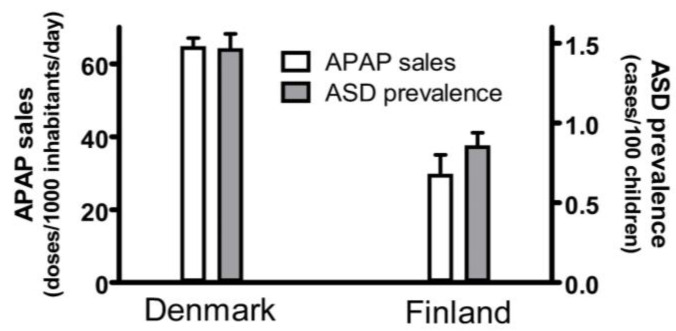
The sales of acetaminophen (APAP) in Denmark and Finland correlated with the prevalence of autism spectrum disorder (ASD) in those countries. The sale of acetaminophen is limited to the sale of drugs containing acetaminophen as the only active ingredient and is measured in units of a “defined daily doses” (equal to 3 g of active ingredient) per 1000 inhabitants per day. The sales for each year from 2006 to 2010, as reported by Wastesson and colleagues [15], were averaged and the mean plotted. Sales for both Denmark and Finland showed upward trends every year, and the upper limit of the range (data from 2010) is shown by the error bars. The prevalence of ASD in Denmark and Finland was reported by Delobel-Ayoub and colleagues [16]. Data are shown for all children, including males and females, born in 2006, with prevalence measured at 9 years of age. The 95% confidence interval reported by Delobel-Ayoub and colleagues is indicated by the error bars.

A current summary of evidence demonstrating that acetaminophen use in susceptible babies and children causes many, if not most, cases of ASD is shown in Table 1. Fourteen associations between acetaminophen use during early neurodevelopment and ASD are evident. Any one geographic or temporal association might be spurious, possibly unrelated to causality. For example, two independent studies, when taken together, show that the popularity of acetaminophen in two Scandinavian countries, Denmark and Finland, correlated with the prevalence of ASD in those countries (Figure 1). First, the sales of acetaminophen per unit population from 2006 through 2010 in Denmark were more than twofold greater than the sales of acetaminophen in Finland during the same time period [15]. Second, for children born in 2006, whose brain development might have been influenced by exposure to acetaminophen between 2006 and 2010, the prevalence of ASD in children born in Denmark was approximately 70% greater than the prevalence of ASD in children born in Finland [16]. Limitations to the conclusions that can be drawn from this previously unreported geographic association are evident. For example, total sales of acetaminophen do not necessarily reflect use of the drug during early development, when induction of ASD is possible, and therefore, this association does not necessarily reflect a direct association between pediatric use of acetaminophen and the prevalence of ASD. Nevertheless, when considered in light of other lines of evidence (Table 1), this association adds to the burden of evidence demonstrating without reasonable doubt that acetaminophen use in susceptible babies and children causes many, if not most, cases of ASD.

**Figure 2:**
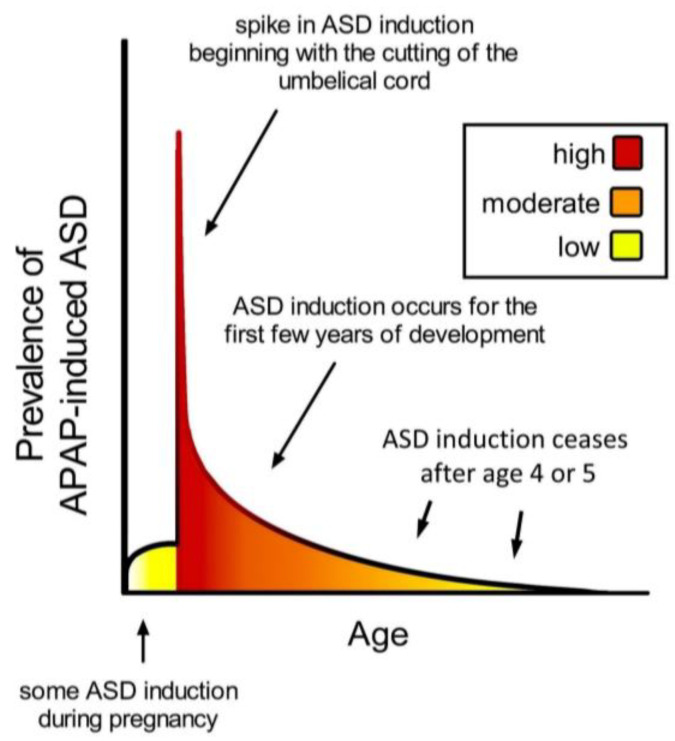
Schematic diagram showing prevalence of acetaminophen (APAP)-induced ASD as a function of age. The neurodevelopmental window of sensitivity is broad, possibly bounded by the first trimester of pregnancy and the end of the 4th or 5th year of life. The shape of the curve is apparently determined by levels of exposure to acetaminophen, and sensitivity to acetaminophen, a function of oxidative stress and ability to metabolize acetaminophen [9]. ASD, autism spectrum disorder; APAP, acetaminophen (paracetamol).

This evidence for a causal relationship between acetaminophen exposure and ASD, when considered in aggregate, is overwhelming [1,2]. However, the question remains, exactly how much of the total burden of ASD is caused by exposure of susceptible babies and children to acetaminophen? The second question that will be addressed in this review is, do the risks of acetaminophen for neurodevelopment outweigh the benefits of the drug as it is currently used?

## 2. Evidence Supports Extensive Influence of Acetaminophen Exposure on Current Rates of ASD

Several observations are consistent with the view that the vast majority of ASD could be induced by acetaminophen. For example, the prevalence of ASD in the 1970s (1/2500), prior to the widespread use of acetaminophen in the pediatric population, was approximately 2% or less of the current prevalence (1/36). Further, a consistent hallmark of ASD is impairment in social awareness, and studies in adult humans indicate that acetaminophen specifically affects social awareness [53,54,67]. Thus, the view that ASD is a hallmark of acetaminophen-induced neurodevelopmental injury is consistent with all available information.

The association of ASD with numerous factors that cause oxidative stress, including but not limited to metabolic abnormalities, some genetic polymorphisms, infections, antibiotic use, and other medical conditions, is consistent with the fact that acetaminophen metabolism is more hazardous in the presence of oxidative stress, a condition associated with risk factors for ASD [9]. Thus, the view that the high prevalence of ASD can be largely accounted for by the currently ubiquitous use of acetaminophen provides a straightforward explanation for the numerous observed risk factors associated with ASD.

An estimation of the relative number of cases of ASD that are induced by acetaminophen might be obtained by considering studies that describe ASD induction during pregnancy, at birth, and later in postpartum development. A compilation of relevant information derived from such studies is shown in Table 2 and is consistent with the view that the vast majority of cases of ASD could conceivably be induced by acetaminophen. During pregnancy, studies of cohort data suggest that some cases of ASD are possibly caused by heavy use of acetaminophen [12,13]. However, based on an assessment of available data, we previously concluded [1] that the number of cases of ASD induced by acetaminophen exposure during pregnancy probably accounts for less than 20% and may account for 10% or less of the total cases of ASD. However, these cases add to the tally induced by postnatal exposure to acetaminophen.

Induction of ASD by acetaminophen near the time of birth apparently contributes extensively to the total cases of ASD induced by acetaminophen. A study in the US found striking associations between ASD and acetaminophen in cord blood [43]. When children were divided into three groups based on their cord blood acetaminophen, the relative prevalence of ASD was 1.0 (baseline), 2.14, and 3.62, positively correlated with acetaminophen levels. Based on these data, one could crudely estimate that cord blood acetaminophen accounts for a 125% increase in ASD, or almost 56% of all cases of ASD {[(1.0 + 2.14 + 3.62)/(1 + 1 + 1)] = 2.2533}. This crude estimate does not consider a number of issues, including potentially confounding factors such as the reason for administration of acetaminophen. However, a 100% (twofold) increase in infantile ASD is associated with circumcision [11], a minor medical procedure often associated with acetaminophen use shortly after birth, supporting the view that the very early postpartum period is a time of very high risk for acetaminophen-induced ASD. As discussed previously [1], a twofold increase in infantile ASD at the time of circumcision could account for 15% to 20% of all cases of autism in a country such as the United States, which has a male circumcision rate of approximately 60% [71]. Finally, the case-controlled study by Schultz [10] found a 20-fold greater prevalence of regressive ASD when acetaminophen was used between 12 and 18 months of age, suggesting that the vast majority of regressive ASD, reflecting approximately 30% of all cases [70], may be due to acetaminophen exposure. The Schultz study included 81 children with ASD and 79 neurotypical controls, but the confidence errors were substantial, leaving uncertainty regarding the exact number of cases of regressive ASD induced by acetaminophen. However, the view that the vast majority of regressive autism might be induced by acetaminophen is supported by surveys of parents with children who have ASD. Between 30% and 50% of parents surveyed in the USA attribute their child’s ASD at least in part to vaccination [51,52], a procedure often accompanied by administration of acetaminophen.

Given the above considerations, the assertion that eliminating exposure to acetaminophen during neurodevelopment would result in a greater than 90% decrease in the prevalence of ASD seems plausible. It could be argued that acetaminophen cannot possibly cause all cases of ASD since ASD was discovered in the 1940s, whereas acetaminophen was not used until the 1950s. However, phenacetin and acetanilide, both of which are converted into acetaminophen by the human body [72,73], were introduced in Germany in the 1880s and widely used as analgesics [73,74] until they were determined to be excessively toxic and their use was banned in most countries in the 1970s and 1980s [73,75]. With this in mind, it is possible that some of the earliest known cases of ASD reported in the 1940s in Germany [76] and in the USA [77] were in fact induced by the metabolite acetaminophen as a result of exposure to phenacetin or acetanilide.

The idea that acetaminophen induces the vast majority of cases of ASD is attractive from a scientific perspective, providing a straightforward explanation for numerous observations in the field, some of which are summarized above. The idea is also appealing from a public health perspective, as it provides a means for rapidly reducing the burden of ASD, which now affects one in every 36 US children [78]. However, the uncertainty (confidence intervals) associated with the Schultz study of regressive autism [10] and with the study of cord blood acetaminophen [43] are very large, and thus, the relative amount of ASD induced by acetaminophen cannot be accurately determined from these studies. Further, studies of large cohorts are complicated by several factors, including poor documentation of over-the-counter acetaminophen use [2], precluding the reliability of the quantitative estimates obtained in such studies [1]. Thus, although the idea that acetaminophen induces the vast majority of cases of ASD is attractive, the idea cannot be embraced with confidence at the present time. However, our perspective is that the induction in most cases of ASD by acetaminophen exposure in susceptible babies and children is likely, and the induction in the vast majority of cases, perhaps more than 90%, is plausible. Without the ability to objectively test the null hypothesis, the historical record provides rationale for this conclusion.

## 3. Alternative Hypotheses

It could be argued that the reason for giving acetaminophen, for example, an infection-induced fever, is a confounding factor. In this hypothetical scenario, the reason for giving acetaminophen is either the cause of ASD induction itself or an indicator of the presence of ASD. However, this explanation does not account for several observations [1,2], including the increasing prevalence of ASD over time and the association of ASD with the circumcision procedure. Further, if infection-induced fevers were the cause of ASD, then it is expected that pandemics associated with unhygienic conditions in the past would have induced pandemics of ASD. Finally, as highlighted above, clinical indications for acetaminophen use generally cause oxidative stress, meaning that the clinical indications precipitating the administration of acetaminophen are not potential confounding factors, but rather are cofactors in the induction of injury.

Environmental factors that cause oxidative stress are expected to enhance acetaminophen-induced neurodevelopmental injury due to their impact on drug metabolism [9]. Thus, the previously noted [9] association of a wide range of oxidative stress-inducing factors with ASD is not surprising. In addition, based on the available evidence outlined in the Introduction (see Table 1), these oxidative stress-inducing factors are not expected to induce ASD in the absence of acetaminophen administration. A notable exception under specific circumstances may be some pesticides. Pesticides in general result in increased oxidative stress [79] and are generally associated with a moderately increased risk of ASD [80]. As such, pesticides fit into a broad category of oxidative stress-inducing factors that negatively impact acetaminophen metabolism and are associated with ASD [9]. However, some pesticides differ from most oxidative stress inducing factors in that they are metabolized by the liver into either acetaminophen [81,82], biologically active metabolites of acetaminophen [83,84], or a variety of compounds structurally related to those metabolites [85]. This suggests the possibility that exposure to some pesticides could induce ASD directly due to the presence of acetaminophen or related metabolites without therapeutic administration of the drug. However, pesticide contaminants in food tend to be less than 1 mg/kg [85,86], and a hundred-fold or more dilution in the human body following consumption and digestion would place the levels of these chemicals hundreds of times lower than the therapeutic dose of acetaminophen, which is approximately 15 mg/kg body weight. Thus, it is expected that typical exposures to pesticides in contaminated food might impose a very minor risk in comparison to therapeutic usage of acetaminophen, and such exposure would not typically, by itself, induce ASD. That being said, heavy exposure during the production, distribution, or use of bulk pesticides could conceivably provide sufficient exposure to acetaminophen or related metabolites for ASD induction in the absence of therapeutic use of the drug. Such a scenario may account for the observation that children of mothers who worked as farmers in Vietnam had an almost fivefold greater prevalence of ASD as compared to children of mothers who were government staff (OR = 4.72, 95% CI 2.03–10.97) [87].

It could be argued that no dramatic change in the prevalence of ASD has occurred over the past 40 years and that much of the perceived change is due to a variety of factors unrelated to neurodevelopment. Proposed factors include changing diagnostic criteria, increasing awareness, and funding/resource-driven increases in diagnosis. However, data pointing at the role of acetaminophen in the induction of ASD undermine this assertion. For example, the much greater prevalence of ASD associated with circumcision [11] and cord blood acetaminophen [43] is difficult to explain if no real increase in the prevalence of ASD exists. While various changes in social and clinical factors undoubtedly caused increases in the measured prevalence of ASD at the time that those factors emerged, careful analysis reveals that such factors do not account for 40 years of steadily increasing prevalence [88]. Further, recent changes in diagnostic criteria tend to reduce, not increase, the diagnosis of ASD: Version Five of the Diagnostic and Statistical Manual of Mental Disorders (DSM-5), published in 2013, is generally considered to provide more, not less, restrictive diagnostic criteria than version four of the manual (DSM-IV) [89], which was published in 1994. Equally important is the consistent ratio through time of severe to mild impairment in individuals with ASD, indicating that changes in prevalence are not due to medical professionals more effectively identifying relatively mild cases. In addition, if there has been little or no real change in the prevalence of ASD in the past century, it is difficult to explain why the condition was not identified until the 1940s and why the measured prevalence did not substantially increase until the 1980s and, particularly, the 1990s. In contrast, if acetaminophen exposure in susceptible babies and children is indeed responsible for a significant portion of ASD, then these trends in time are fully expected.

Because of its association with inflammation [9,90,91] and its currently increasing prevalence, ASD fits into a category of now-prevalent medical conditions that emerged following the second industrial revolution [92,93]. Those conditions, generally associated with increased inflammation, include allergies, autoimmune diseases, some neuropsychiatric disorders, and a variety of cardiovascular problems [94,95,96]. Rare or even absent in ancestral human populations, these conditions emerged as a result of environment and/or lifestyle changes [94,95,96]. Thus, the dramatically increased prevalence of ASD fits into an established pattern among numerous medical conditions, and its dramatically increased prevalence can be taken as an indication of environment and/or lifestyle changes, not as an indicator that a common condition was previously mistaken as rare. Indeed, dozens of rare, mutation-induced diseases have been identified over the past two centuries [97], and the prevalence of those diseases has remained rare.

## 4. Periods of Sensitivity to Acetaminophen during Neurodevelopment

The timing of acetaminophen-mediated induction of ASD during brain development is of considerable interest. Based on an analysis of cohort data by Liew and colleagues [12], the neurodevelopmental window sensitive to acetaminophen may begin very early during pregnancy, perhaps in the first trimester (Figure 2). However, the time of birth is evidently the most critical period in terms of sensitivity to acetaminophen-induced neurodevelopmental injury (Figure 2). The view that newborns are exquisitely sensitive to acetaminophen is consistent with the 2-fold greater prevalence of infantile ASD associated with circumcision [11] and the 3.6-fold greater prevalence of ASD in the third of children with the highest levels of acetaminophen in their cord blood compared to the third with the lowest levels [43]. As discussed previously [2], the well-established age-dependent pharmacology of acetaminophen [98,99] dictates that, by unit weight, the mother/fetus dyad is far more capable of metabolizing and detoxifying acetaminophen than the newborn. The dramatic differences in weight-adjusted capacity to metabolize and detoxify acetaminophen between pregnant women and newborns [2] are expected to adversely affect the developing brain of susceptible newborns when the umbilical cord is cut in the presence of acetaminophen. Based on pharmacokinetic considerations, the first 10 days of life, in particular, should be the most sensitive to acetaminophen, when the glucuronidation process, important for the detoxification of acetaminophen [2], is not yet functional [100]. Following the perinatal period, the work by Schultz [10] discussed above and the observations from parents discussed previously [9] indicate that acetaminophen induces many, if not most, cases of regressive ASD. Based on a meta-analysis by Tan and colleagues [70], regression can occur in children as old as 4 or 5 years, but the time distribution of regression is skewed toward earlier ages, with half of all cases of regression probably occurring before approximately 1.5 years of age. Thus, as shown in Figure 2, the neurodevelopmental window for the induction of ASD by acetaminophen is broad, with the prevalence of induction apparently dictated by the level of drug exposure and by susceptibility.

The current presumption in the medical community that acetaminophen is safe for pediatric use is based on dozens of studies in babies and children that incorrectly assumed that the toxic effects of the drug in adults would be the same for babies and children [32]. However, given that the therapeutic target of acetaminophen involves brain function, pediatric use of acetaminophen should have been preceded by extensive tests using animal models for neurodevelopment [1]. Preclinical toxicity screens using laboratory animals often fail to detect drug toxicity in humans [101]. However, neurodevelopment is a conserved process across mammalian species, and laboratory animals provide a very good model for examining brain sensitivity in the perinatal period [102]. Indeed, the toxicity of acetaminophen for the developing brain *is* readily detected using perinatal laboratory animal models [1,3,4,5,6,7,8]. Given the severe adverse effects of acetaminophen on neurodevelopment observed in laboratory animals by several independent laboratories using a variety of experimental designs, we conclude that the standard safeguards involving preclinical testing that protect the population from exposure to drugs with a poor benefit-to-risk ratio were not employed when acetaminophen was introduced into widespread pediatric use in the 1980s.

Based on available evidence (Table 1), we are confident that many cases of ASD, and perhaps the vast majority, are caused by exposure of susceptible individuals to acetaminophen during early stages of neurodevelopment. With this in mind, we urgently encourage regulatory and policy-making bodies to restrict the pediatric use of acetaminophen and stress the importance of consumer education to inform individuals of current knowledge regarding the impact of acetaminophen on the developing brain. The fact that consumers lack education regarding the dangers of acetaminophen for neurodevelopment is evident from numerous studies outlined below showing widespread frivolous use of the drug for lowering body temperatures that are not actually high enough to be classified as a fever [103,104,105,106,107] and misuse of the drug either by giving too high of a dose or administering the drug too frequently [105,106,107,108,109,110,111,112,113,114].

## 5. Improper Use of Acetaminophen to Treat Fevers Is Common

Existing studies suggest that acetaminophen is not administered to children in a manner that weighs the drug’s evident benefits against its risks, resulting in an overaggressive administration of antipyretics in children. Part of this problem involves misconceptions regarding what temperature constitutes a fever. According to the definition first described by Carl R.A. Wunderlich more than a century ago [115] and still widely accepted today [116], a fever in humans constitutes a temperature greater than or equal to 38.0 °C (100.4 °F). However, a temperature of 38.3 °C (100.9 °F) is a more appropriate cutoff for a fever [117], with many healthy infants having a normal temperature of 38.1 °C or 38.2 °C, especially during the summer months [118]. Nevertheless, approximately half of parents consider a temperature of less than 38 °C (100.4 °F) to be a fever [119], and among surveyed pediatric emergency nurses, 46% also stated that a temperature less than 38 °C is considered a fever [120]. These findings indicate that many parents and health care workers do not know how to accurately define a fever.

Fever in a variety of circumstances, including brain injury, is associated with worsened outcomes and can lead to damage to specific organs, including the kidneys and the liver [121]. However, fevers associated with infection constitute a critical component of the immune response to infection and are beneficial [122,123]. Evans and colleagues, for example, assert that an increase of 1 to 4 °C in core body temperature is associated with “improved survival and resolution of many infections” [124]. With this in mind, antipyretic treatments are, in fact, immunosuppressive. Further, even within the higher range of 40 °C to 42 °C, there is no evidence to suggest that typical fevers in children without brain injury present an increased risk for adverse health outcomes such as brain damage [116,122,123].

Several investigators have reported “fever phobia”—exaggerated concerns about fever in children and its complications (e.g., seizures, brain damage, etc.) [106,116,119,122,125]. Ninety-one percent of caregivers believe fevers can have harmful effects, with 21% of caregivers listing brain damage and 14% listing death [119]. Further, sixty percent of pediatricians state that temperatures of 104 °F (40 °C) or greater can cause seizures, brain damage, or death [104]. However, as stated by the American Academy of Pediatrics, to their knowledge, a child dying from a simple febrile seizure “has never been reported” [126]. This is despite the fact that 2–5% of all children experience febrile seizures. Fever in children causes disproportionate anxiety even among health care professionals; for example, among pediatric emergency nurses, 38% state that temperatures less than 40 °C could cause serious complications [120].

Consequentially, antipyretics are administered by caretakers [106,116,119,125] and pediatric health care professionals [104,120], even when there is minimal fever or no fever. A survey of 340 caregivers in two hospital-based pediatric clinics in Maryland found that 25% of caregivers gave antipyretics for temperatures under 37.8 °C (less than 100 °F) [119]. Another survey of 230 caregivers of children in a Pediatric Emergency Department in Virginia reported that 63.9% considered a temperature of less than 37.8 °C to be the minimum temperature for antipyretics [106]. A survey of caregivers of 201 children in Israel estimated that 65.2% of caregivers indicated that they would administer antipyretics for temperatures lower than 38 °C [105]. Further, among pediatricians in Massachusetts, 72% reported they always or often recommended treatment to reduce fever (including acetaminophen), and 89% stated they did so at temperatures between 38.3 °C and 38.9 °C [104]. Finally, an Italian study found that a surprising 74% of all administrations of acetaminophen for fever were given to treat fevers less than 38.4 °C. The authors conclude that “preventive action should be taken regarding the use of acetaminophen as antipyretic drug in children in order to reduce the fever phobia and self-prescription…” [103]. Thus, with the possible exception of a study of 402 parents in Palestine that found that only 1.5% would give antipyretics for temperatures less than 38 °C [125], numerous studies point toward a wide-spread fever phobia, with many parents and even health care workers overtreating fevers.

## 6. Overdoses of Acetaminophen in the Pediatric Population

Acetaminophen has a relatively low “therapeutic index”—the difference in the amount required for a therapeutic effect and the amount that is toxic is relatively small. A low therapeutic index, coupled with wide availability and apparently wide use, poses safety concerns with respect to dosing [127]. Research indicates that some caregivers administer incorrect doses to children, with some studies demonstrating a supratherapeutic dosage being given [105,109,110,113,114] and other studies finding administration at intervals that are too frequent [105,106,108,109,110,111,112]. Additionally, the observation that more than one medication containing acetaminophen is being given to children at the same time, resulting in overexposure to the drug, is also problematic [128].

A variety of evidence indicates that overdoses of acetaminophen in the pediatric population are common. A study of caregivers to 200 children in Turkey, for example, found that 8.4% of the patients received doses of acetaminophen that exceeded the recommended maximum dose [113]. Further, a study of another 200 patients aged 10 years or younger at the pediatric emergency department at Jacobi Medical Center in New York found that 15% of 124 patients receiving acetaminophen were given overdoses [109]. The authors also noted that a combined 51% of caregivers incorrectly stated that the dosage should be based on either the age of the child or the height of the fever; caregivers who correctly stated that the dosage should be based on their child’s weight were significantly less likely to give the wrong dosage (relative risk (RR) = 0.71, *p* < 0.03, 95% CI = 0.52–0.97) [109]. In another Turkish study, 12.1% of parents overdosed their child with acetaminophen [114]. A study in Saudi Arabia estimated that 27% of children aged 14 or younger who had been given acetaminophen for fever prior to visiting the emergency department were given a supratherapeutic dose of acetaminophen [110]. Similar results were found in an Italian study, with 24% of children visiting a primary care center for fever having received an overdose of acetaminophen [103]. In one of the most dramatic examples of overdosing, among 201 caregivers surveyed in Israel, 34.8% reported administering higher-than-recommended doses of acetaminophen [105].

In addition to overdose of acetaminophen via administration of too much drug, as described above, studies from around the world point toward all-too-common administration of a greater number of doses within a given time frame than is recommended. For example, an Australian survey of 401 parents found that 3.8% reported intervals of administration to their children that were too short—medication was administered at intervals shorter than the accepted minimum of 4 h [108]. A similar study conducted in New York found that 4% of caregivers administered acetaminophen to children too frequently [109]. Furthermore, a survey in Baltimore found that, among 340 caregivers, 14% gave acetaminophen to children every 3 h or less [119]. In another study, this one in Virginia, 8% of 230 caregivers administered the drug to children too frequently [106]. A Saudi Arabian study found that 14% of caregivers administered acetaminophen to children too frequently [110]. Twenty-seven percent of caregivers surveyed in Abu Dhabi, United Arab Emirates, reported giving their child acetaminophen more frequently than every 4 h [111]. Among 201 children in Israel, 19.9% were given acetaminophen every 1–3 h if their fever persisted [105]. Further, a retrospective study showed that 52% of pediatric patients with hepatotoxicity had received adult preparations of acetaminophen [112].

Another possible facet of acetaminophen overdose is administering different medications that contain acetaminophen. A survey conducted by Princeton Survey Research Associates International in 2013 found that 35% ± 6.7% of the parents amongst the 1003 adults surveyed said it was safe to administer the maximum dosage of Children’s Tylenol^®^ in combination with Children’s Tylenol Plus Multi-Symptom Cold^®^ to a child [107]. Considering that both products contain acetaminophen, this could lead to a dose of acetaminophen that is toxic for children [127,128,129].

The problems with misuse of acetaminophen described above are numerous and include administration of a dose that exceeds the maximum recommended dose, too frequent administration of the drug, simultaneous administration of more than one formulation containing the drug, and administration of the drug for invalid reasons. Given the frequency of these problems, as described above, it is apparent that the risks of administering acetaminophen are not seriously considered by many caregivers. One potential indicator of the casual indifference toward exposure of the developing brain to acetaminophen is the treatment of infants during circumcision without regard for the amount of acetaminophen that might be carried over from their mother following birth. Given the apparent importance of cord blood acetaminophen and acetaminophen administration during circumcision in the induction of ASD, described above, this potential facet of acetaminophen overdose should not be overlooked. This potential method of exceeding the recommended dose of acetaminophen adds to the other problems described above, all of which violate the currently accepted standards and reflect widespread disregard for neurodevelopmental risks associated with pediatric use of acetaminophen.

## 7. Misguided but Currently Accepted Use of Acetaminophen

Some immediate changes in currently accepted uses of acetaminophen are warranted because the accepted practice is not justified based on available evidence. Although acetaminophen, when administered rectally in a hospital setting, may prevent some repeated febrile seizures during a single febrile episode [130], most controlled studies find that acetaminophen does not prevent febrile seizures in pediatric patients [131]. This conclusion indicates that acetaminophen is generally not helpful under circumstances that are often assumed to be dire and for which the drug is often assumed to be essential. However, a steering committee for the American Academy of Pediatrics points out that “with the exception of a high rate of recurrence, no long-term adverse effects of simple febrile seizures have been identified” [126]. Thus, the view that a simple febrile seizure poses a significant risk is an assumption that is contradicted by studies that have attempted but failed to identify any such risk [132,133,134].

For three decades now, the effect of acetaminophen on febrile seizures has been questioned. Schnaiderman and colleagues at Tel Aviv University concluded in 1993 that “prophylactic administration of acetaminophen in children with febrile seizures is not effective in the prevention of fever, the reduction of its degree, or in preventing the early recurrence of febrile seizures” [135]. In their study, Schnaiderman evaluated 104 children, with an average age of 1.9 years, who were admitted to the hospital for a febrile seizure. Prevention of a subsequent febrile seizure was attempted by administration of acetaminophen every 4 h for four days or until the body temperature returned to normal for 12–18 h. As a control group, children were treated with acetaminophen “as needed” when their body temperature reached 37.9 °C, which is approaching the minimum temperature defined as a fever, 38.0 °C (100.4° F). This approach resulted in substantially greater amounts of acetaminophen being administered to the group given prophylaxis, but without a significant effect on outcome. However, Schnaiderman did not use a placebo group and acknowledged that lower levels of acetaminophen may have resulted in more febrile seizures. Nevertheless, Schnaiderman was able to conclude confidently that additional acetaminophen would not have been helpful. This conclusion is important, as it points toward the futility of administering excess acetaminophen in an attempt to bring down fevers.

After the Schnaiderman study, Uhari and colleagues at the University of Oulu, using a placebo group, conducted a study designed to test the potential for acetaminophen to prevent febrile seizures. In 1995, they concluded that treating fevers 40 °C or higher with acetaminophen had no effect on the occurrence of febrile seizures [136]. Uhari’s study involved a two-year follow-up time with 153 children who had previously experienced a febrile seizure, and the average age was approximately 1.7 years at the beginning of the study. Over a decade later, evaluating a different cohort of children, investigators at the University of Oulu confirmed the results from their earlier study, concluding that antipyretic agents, including acetaminophen, “are ineffective for the prevention of recurrences of febrile seizures and for the lowering of body temperature in patients with a febrile episode that leads to a recurrent febrile seizure” [137]. This conclusion from the University of Oulu was subsequently supported by others [122,138,139,140], including a steering committee for the American Academy of Pediatrics [126]. However, Murata and colleagues recently reported that rectal administration of acetaminophen in a hospital setting can prevent approximately 60% of febrile seizure recurrences within the same febrile episode when compared to children who were not treated (no placebo) [130]. Unfortunately, Murata concluded that the drug is “safe” under these circumstances, not taking into account the significant risk the drug poses to neurodevelopment [1,2] and the apparent non-risk of febrile seizures [126].

In addition to the discontinued use of acetaminophen for the treatment of fevers in the pediatric population, acetaminophen should no longer be used to treat pain and discomfort in cases where it is proven to be ineffective for such treatment. For example, a controlled study demonstrated that acetaminophen does not block the pain of circumcision [141]. Further, despite the fact that the use of acetaminophen before and after vaccination is common [142], the World Health Organization does not recommend routine acetaminophen use with vaccination [143] because it may dampen the intended effects of the vaccines [144] and/or because it has not proven to be effective for management of the pain and discomfort that can accompany vaccination.

The lack of a beneficial effect of acetaminophen on some important outcomes is probably not unique to the pediatric population. A randomized, controlled clinical trial showed that treatment of infection-associated fevers with acetaminophen in hospitalized adults did not improve mortality rates or the need for intensive care [145]. The patients in this study experienced approximately 16% mortality during the 90-day study period, and were, on average, undoubtedly less healthy than the average individual with a fever who is not hospitalized. This study therefore indicates that, even under dire circumstances, treatment of infection-associated fevers in adults is not warranted. Furthermore, fevers are an important and effective component of the immune response against infection [116,122,123,124,146]. As such, any effort to block the benefits of a fever should be justified by evidence that the effort carries benefits that outweigh the risks. Thus, current evidence indicates that the benefits of reducing fevers with acetaminophen are unproven at best and possibly non-existent, whereas the risks of the treatment in the pediatric population regarding neurodevelopment are definitely considerable.

## 8. Changes in Practice Need to Be Made

Recommended changes to established obstetric and pediatric practices can be classified into five categories based on current recommendations from governing medical organizations and on current evidence regarding benefits of treatment. Importantly, changes in four of the five categories are expected to have minimal or no negative impacts on medical management of patients.

Category 1: Administration of acetaminophen in a manner that was never intended should be discontinued. This includes treatment of temperatures that do not technically constitute a fever and administration of the drug more frequently and at higher doses than recommended.
Category 2: Administration of acetaminophen under conditions in which evidence demonstrates a lack of effectiveness should be discontinued. This includes the treatment of the pain of circumcision and perhaps the treatment of fevers to prevent febrile seizures.
Category 3: Administration of acetaminophen under conditions in which no evidence demonstrates long-term benefits of treatment or in which evidence demonstrates a lack of long-term benefits should be discontinued. This includes the treatment of fevers and prophylactic treatments prior to labor and delivery.
Category 4: Administration of acetaminophen that is no longer recommended by governing medical bodies should be discontinued. This includes the treatment of patients receiving vaccinations and will hopefully include many more reasons for administration in the future.
Category 5: Administration of acetaminophen under conditions where evidence indicates that it is or may be beneficial should not be continued without disclosure of the drug’s long-term risks for neurodevelopment. All caregivers, including parents, should be made aware of evidence related to both benefits and risks so that they can make informed decisions.

Some of the above-recommended changes to pediatric practice should have immediate beneficial effects with little to no decrease in medical care. Most obviously, discontinuation of the misadministration of acetaminophen (Category 1) is expected to prove useful and may be facilitated by public education regarding the risks of the drug regarding neurodevelopment. Public education, while likely necessary to accomplish the suggested Category 1 changes, may simultaneously facilitate the recommended Category 5 changes. As another example, given the apparently heightened sensitivity of the brain to acetaminophen-mediated injury during the neonatal period (Figure 2), eliminating the use of acetaminophen for circumcision (Category 2) and for the hepatitis B vaccine (Categories 3 and 4), two situations for which acetaminophen is often administered in the first hours of life, could make a significant difference in the prevalence of ASD and is expected to have minimal adverse consequences.

Perhaps the most difficult decisions by caregivers will need to be made for acetaminophen use in Category 5, where evidence indicates that acetaminophen is or perhaps might be helpful. Indications that fall under this category include pain management under some conditions. Studies, which are generally conducted with adults rather than children [147,148], show that acetaminophen mitigates pain under a range of conditions and that the drug has been widely accepted as an effective analgesic [149]. However, the value of pain management using acetaminophen is being questioned [149], with the benefits of the treatment being relatively minor in some scenarios. For example, a metanalysis of randomized controlled trials for the treatment of chronic arthritis pain showed that the relative percent improvement with treatment using acetaminophen was only 5% from baseline, with an absolute change of 4 points on a scale of 0 to 100 [150]. Similarly, in a metanalysis of randomized controlled trials for the treatment of post-bariatric surgical pain, the authors concluded that the use of acetaminophen after bariatric surgery effectively reduced pain scores after 24 h and effectively reduced the use of opioids [151]. However, as the authors point out, “*While the present study has found that IV acetaminophen leads to a statistically significant decrease in pain score of 0.66 (95% CI 0.28 to 1.03) calculated using a 10-point VAS* (Visual Analogue Scale), *the results are not definitively higher than the minimally clinically important difference (MCID) associated with pain which ranges from 0.8 to 4 on a 10-point scale. Furthermore, while this review found an opioid-sparing effect of 6.44 mg in MED* (morphine equivalent doses), *this translates to less than a 10% reduction in morphine used*” [151]. Thus, the presumed benefits of analgesia using acetaminophen should be critically evaluated rather than taken for granted by caregivers, keeping in mind the risks to neurodevelopment during early brain development.

It is within clinical contexts involving Category 5 use of acetaminophen that regulatory agencies and physician associations need to carefully evaluate guidelines, considering the benefits and risks of acetaminophen and of alternative pharmacological and non-pharmacological-based management strategies for pain and discomfort. In addition, more research is urgently needed to find safe alternatives. We had previously hypothesized that intravenous acetaminophen, a preparation of acetaminophen containing antioxidants known to counteract the toxic effects of acetaminophen poisoning, would be safe for neurodevelopment. However, even at doses lower than the acceptable dose currently used in humans, repeated administration of the drug mixture to laboratory rats during the neonatal period resulted in dramatically increased anxiety later in life compared to controls [6]. It could be argued that lowering the dose of acetaminophen might eliminate adverse effects on neurodevelopment. However, lowering the dose of the drug by 50% dramatically reduces the effectiveness of the drug [152]. With this in mind, based on laboratory animal studies, there is no known safe and effective dose of acetaminophen during early periods of brain development at the present time. This does not, however, rule out the potential for future development of acetaminophen-based therapeutics that effectively avoid any adverse neurodevelopmental consequences.

Change can and does happen. An advisory panel for the US Food and Drug Administration (FDA) recently determined that oral phenylephrine at the currently recommended dose is ineffective at relieving nasal and sinus congestion [153]. This determination is quite remarkable because oral phenylephrine has been considered safe and effective since the 1970s and is an active ingredient in a wide range of popular medications, including Sudafed PE^®^, Tylenol Cold^®^, Alka-Seltzer Plus Cold^®^, and numerous Mucinex^®^ formulations. As a result, the world’s largest healthcare company, CVS Health Corporation, will no longer sell products with phenylephrine as the sole active ingredient. It is time for another major and far more important change, as regulatory agencies, academies of obstetricians, and academies of pediatricians are encouraged to acknowledge the profound dangers of acetaminophen during neurodevelopment and the lack of convincing data demonstrating long-term benefits from pediatric use of the drug during sensitive periods of brain development.

## Figures and Tables

**Table 1:** Current evidence leading to the conclusion that early exposure of individuals to acetaminophen (APAP) causes many, if not most, cases of ASD. Twenty-two lines of evidence, with 20 independent lines of evidence, are provided. Components of lines of evidence numbers 12 and 13 are derived from the same sources measuring temporal trends in ASD prevalence. Thus, although multiple sources corroborate those temporal trends, those two lines of evidence are not entirely independent. Lines of evidence 16 and 17 are derived from the same case-controlled study involving 81 individuals with ASD, and thus, those two sources are not independent.

Summary of Evidence	Nature of Evidence
1. Early life exposure to APAP at doses similar to or even less than doses received by human babies and children results in long-term, profound modification of brain function in both laboratory mice and rats [5,6,7,8,17], by definition a severe adverse event that should have precluded any clinical testing of APAP in babies and small children.	Multiple, independent laboratory animal studies demonstrate that APAP is not safe for neurodevelopment.
2. In laboratory rats, APAP affects the developing male brain more than the female brain [8]. In laboratory mice, males are more susceptible to APAP-mediated liver injury than are females [18]. ASD is more prevalent in males than in females [19].	Laboratory animal studies of APAP-mediated injury reflect the sex distribution of ASD in humans.
3. APAP causes apoptosis-mediated death of cortical neurons in adult laboratory rats at concentrations lower than it causes liver failure [20]. Affected cortical neurons are implicated in ASD [21,22], and individuals with ASD have increased levels of biomarkers for neuronal apoptosis [23,24,25].	A laboratory animal study of APAP-mediated brain injury reflects biomarkers of injury in humans with ASD.
4. Adult cats are susceptible to APAP-mediated injury due to the lack of a robust glucuronidation-dependent capacity for metabolism [26,27,28,29]. Human neonates similarly lack a robust glucuronidation-dependent pathway [30,31].	Metabolic status causing sensitivity to APAP-mediated injury in an animal model reflects the metabolic status of human neonates.
5. APAP use in babies and children was assumed to be safe during the 1970s despite the fact that it targets brain function and was never shown to be safe for neurodevelopment [32].	Demonstration that the current safeguards for drug approval were bypassed for pediatric use of APAP.
6. Circumcision of males, often performed using APAP as an analgesic, is associated with a twofold increase in the risk for early-onset (infantile) ASD [11].	Temporal association with neonatal APAP use and ASD.
7. APAP-containing products used by South Korean children were repeatedly found to contain amounts of the drug, exceeding the package label [33], and an exceptionally high prevalence of ASD was identified in South Korea [34,35].	Temporal association of accidental, excess APAP administration and ASD.
8. The popularity of APAP use and the prevalence of ASD was substantially higher in Denmark than in Finland in the mid-2000s (Figure 1).	Geographic association between APAP use and ASD.
9. Ultra-Orthodox Jews [36] and Arabs [36,37] in Israel have a reported prevalence of ASD less than half of that of other Israelis. Traditional circumcision practices employed by Ultra-Orthodox Jews do not utilize APAP, and circumcision practices in Arab communities take place outside of the neurodevelopmental window sensitive to ASD induction (Figure 2).	Temporal association between neonatal use of APAP and ASD.
10. Analysis of 61,430 babies in the Danish National Birth Cohort found an odds ratio (OR) of 1.3 (CI 1.02–1.66) for ASD associated with postnatal APAP exposure [13]. The approach used in the analysis is expected to dramatically underestimate the real odds ratio [1].	Association with postpartum APAP use and ASD from one epidemiologic study.
11. The ratio of regressive to infantile ASD rose at the same time as pediatric APAP use rose [14] after aspirin was associated with Reye’s syndrome [9].	Temporal association between the pediatric use of APAP and the qualitative nature of ASD.
12. The incidence of ASD began to increase in the early 1980s, coinciding with the increase in APAP use after aspirin was associated with Reye’s syndrome [9].	Temporal association between pediatric use of APAP and the prevalence of ASD in the early 1980s.
13. The incidence of ASD has steadily increased [9] as direct-to-consumer advertising [38] and perhaps other factors have driven up the use of pharmaceutical products.	Temporal association between the use of APAP and the prevalence of ASD post-1990.
14. Maternal use of APAP during pregnancy is associated with long-term effects that include lower IQ, increased ASD, and increased ADHD in their children [12,13,39,40,41,42,43,44,45,46,47,48,49,50].	Association with prepartum APAP use and neurodevelopmental problems from numerous epidemiologic studies, some with controls for indication.
15. Levels of APAP in cord blood are associated with ASD [43].	Association with APAP use during the peripartum period and the prevalence of ASD.
16. APAP given alongside the MMR vaccine but not the MMR vaccination alone was associated with ASD [10].	Dramatically enhanced risk of ASD associated with the use of APAP with vaccination found in a case-controlled study involving 81 children with ASD.
17. APAP use during early childhood is associated with a dramatic increase in regressive ASD [10].	Dramatically enhanced risk of regressive ASD associated with APAP use found in a case-controlled study involving 81 children with ASD.
18. Many parents believe that their children’s ASD was induced by a vaccine [51,52]. APAP is frequently used with vaccinations, although vaccinations alone do not cause ASD.	Association between APAP use and ASD inadvertently and consistently made by a substantial fraction of parents of children with ASD.
19. APAP use in adults temporarily blunts social trust [53] and awareness [54], emotional responses to external stimuli [55], and the ability to identify errors [56], indicating that the drug targets regions of the brain affected in patients with ASD.	The transient effects of APAP in adult humans are reflected in the symptoms of ASD.
20. Cystic fibrosis is associated with unusually efficient (effective) metabolism of APAP [57,58], and evidence suggests that the prevalence of ASD is very low in patients with cystic fibrosis [9].	Resistance to APAP-mediated injury is apparently associated with a very low prevalence of ASD.
21. Genetic and immune factors associated with an increased risk of ASD have a detrimental effect on the body’s ability to metabolize APAP [9,59,60].	Plausible mechanism: risk factors for ASD and for adverse reactions to APAP are equivalent.
22. APAP is known to be highly toxic in the presence of oxidative stress [61] via a mechanism that involves the formation of the toxic metabolite NAPQI [62,63,64] and concomitant mitochondrial damage [65]. Oxidative stress [9] and possibly mitochondrial dysfunction [66] also play a role in ASD.	Plausible mechanism: production of toxic metabolites from APAP under conditions involved with ASD pathology is established.

**Table 2:** Crude estimates of the relative fraction of total ASD induced by acetaminophen (APAP) at different times during neurodevelopment. The “early postnatal period” is defined for these purposes as the first 5 days postpartum, when many neonates would have been circumcised [68] prior to leaving the hospital [69]. The time when regression becomes observable is unclear [70], and therefore, the time between the peripartum period and when regression is observable is given as a range, between 2 and 12 months. A paucity of information exists for the period after the early postnatal period until regressive ASD would potentially be observable, between 2 and 12 months of age. ASD, autism spectrum disorder.

Time Period (Age)	Crude Estimates of Relative Fraction of Total ASD Induced by APAP	Source of Data/Information for Crude Estimate
Prenatal	10–20%	Cohort studies
Early postnatal period (birth to 5 days)	50–60%	Association of ASD with cord blood acetaminophen [43], supported by association of ASD with circumcision [11]
5 days until 2–12 months (regression not observable)	No information available	Not applicable
2–12 months until 4 or 5 years (regression observable)	20–30%	Small case control study [10], supported by observations of parents [51,52]

## Data Availability

No new data were created or analyzed in this study. Data sharing is not applicable to this article.
